# Homozygous 6-bp deletion of *IGFALS* in a prepubertal boy with short stature

**DOI:** 10.1038/s41439-024-00285-w

**Published:** 2024-07-26

**Authors:** Hibiki Doi, Ikuko Kageyama, Yuko Katoh-Fukui, Atsushi Hattori, Maki Fukami, Naoto Shimura

**Affiliations:** 1 Department of Molecular Endocrinology, National Research Institute for Child Health and Development, Tokyo, Japan; 2 Department of Advanced Pediatric Medicine, Tohoku University School of Medicine, Tokyo, Japan; 3 Department of Pediatrics, Tokyo Rinkai Hospital, Tokyo, Japan

**Keywords:** Medical genetics, Growth disorders

## Abstract

Biallelic *IGFALS* variants lead to acid‒labile subunit (ALS) deficiency characterized by growth hormone resistance with or without delayed puberty. Here, we report a prepubertal boy with a homozygous 2-amino acid deletion within the fourth N-glycosylation motif (c.1103_1108del, p.N368_S370delinsT) associated with parental consanguinity. He showed short stature consistent with ALS deficiency. This case expands the mutation spectrum of *IGFALS* to include the elimination of only one N-glycosylation motif of ALS.

*IGFALS* on 16p13.3 encodes the acid-labile subunit (ALS). ALS forms a ternary complex with insulin-like growth factors (IGFs) and IGF-binding proteins (IGFBPs) and thereby regulates the half-life of IGFs in the circulation^1^. ALS plays a critical role in the growth-promoting effects of growth hormone (GH)^1^. The ALS protein contains seven N-glycosylation motifs with a consensus 3-amino acid sequence (N-X-S/T)^2–4^. These motifs mediate the formation of the ternary complex^2–4^. To date, more than 60 patients with ALS deficiency resulting from biallelic *IGFALS* variants have been reported^1^. Most of these patients exhibited moderate short stature due to GH resistance with or without delayed puberty, although several patients achieved low‒normal adult height^1,5,6^. Salient endocrine findings of these patients were low levels of IGF-I and IGFBP-3 and normal or elevated levels of GH^1,5^. Known *IGFALS* variants associated with ALS deficiency include several substitutions and indels widely distributed in the coding region (Fig. 1)^1,6–18^. However, only two of these variants (p.S87L and p.N580del) caused changes in the consensus amino acid sequence of the N-glycosylation motifs^7,16^. The pathogenicity of these two variants remains uncertain. This is because the p.S87L variant was coupled with an additional variant in *IGF1R*, and the p.N580del variant was present only in a heterozygous state^7,16^. In fact, Janosi et al. reported that site-directed mutagenesis to replace the asparagine residue at any of the N-glycosylation motifs with an alanine residue did not alter the in vitro binding activity of ALS to the IGF/IGFBP binary complex^2^.

**Fig. 1:**
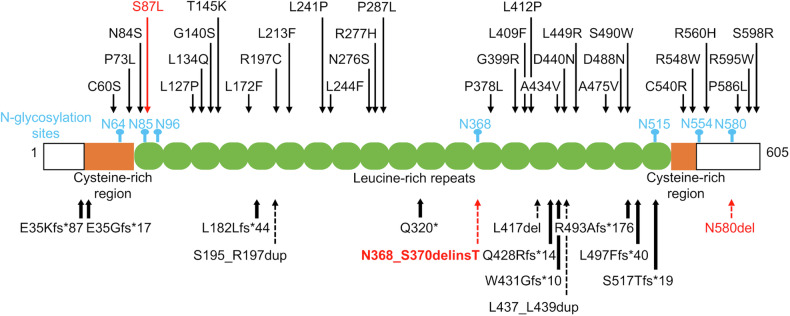
Major *IGFALS* variants identified in patients with short stature. Missense substitutions, indels, and protein-truncating variants are indicated by thin, broken, and thick arrows, respectively. The seven N-glycosylation sites are shown in blue. Variants within N-glycosylation motifs are shown in red. The 2-amino acid deletion in the present case is boldfaced. Brown boxes and the green structure indicate cysteine-rich regions and leucine-rich repeats, respectively.

Here, we report a patient who carried a homozygous 2-amino acid deletion within the fourth N-glycosylation motif of ALS. The patient was a Japanese boy born from a first-cousin couple at 39 weeks and 5 days of gestation. His body weight and length at birth were 2342 g (−2.6 SD) and 47 cm (−1.2 SD), respectively. During childhood, his growth pattern followed the −2.5 SD growth curve of Japanese boys. At 5.2 years of age, he visited our clinic for the evaluation of short stature. Physical examinations showed no phenotypic abnormalities except for short stature (96.6 cm, −2.7 SD). He had no signs of pubertal sexual development. His bone age was 1.5 years younger than his chronological age. Endocrine examinations revealed high basal levels of GH (8.6–22.5 ng/ml; reference range, ≤ 2.5 ng/ml) and a low IGF-I level (17 ng/ml; reference range, 44–193 ng/ml). Arginine stimulation yielded a prominent GH response (82.0 ng/ml; reference range, ≥ 6.0 ng/ml). The levels of thyroid hormones and gonadotropins were within the reference ranges for age-matched boys. The heights of his father and mother were 165 cm (−1.0 SDS) and 148 cm (−1.9 SDS), respectively. The height of his 9-year-old sister was −2.0 SDS.

We performed molecular analyses. This study was approved by the Institutional Review Board Committee at the National Center for Child Health and Development. Written informed consent was obtained from the parents of the patient. Genomic DNA samples were obtained from the peripheral leukocytes of the patient and his parents. We performed mutation screening for 16 major growth genes (*ACAN, CDKN1C, FGFR3, GH1, GHR, GHRHR, GHSR, GNAS, IGF1, IGF1R, IGF2, IGFALS, JAK2, NPR2, SHOX*, and *STAT5B*) using a next-generation sequencer panel (Kazusa DNA Research Institute, Kisarazu, Japan). The methods were described previously^16^. As a result, we identified a homozygous 6-bp deletion (NM_004970.3: c.1103_1108del, p.N368_S370delinsT) in exon 2 of *IGFALS*. No pathogenic variants were detected in the other genes examined. Sanger sequencing confirmed that the patient had a homozygous *IGFALS* variant, and his parents were heterozygous carriers (Fig. 2a). The *IGFALS* variant was not found in the Human Genome Mutation Database (https://www.hgmd.cf.ac.uk/ac/index.php), ClinVar (https://www.ncbi.nlm.nih.gov/clinvar/), the Genome Aggregation Database (https://gnomad.broadinstitute.org/), or the Japanese Multi Omics Reference Panel (https://jmorp.megabank.tohoku.ac.jp). This variant was classified as a variant of uncertain significance (VUS) based on the American College of Medical Genetics and Genomics/Association for Molecular Pathology guidelines (PM2, PM4, PP4)^19^. This variant caused a 3-amino acid deletion and a 1-amino acid insertion in the fourth N-glycosylation motif of ALS (Fig. 2a). Comparative genomic hybridization of the patient using a CGH + SNP array (SurePrint G3 Human Genome Microarray, 2 × 400 k format; Agilent Technologies, Santa Clara, CA, USA) detected loss of heterozygosity (LOH) regions on 10 different chromosomes (Fig. 2b). The total size of the LOH regions was ~187 Mb. The LOH region on chromosome 16 encompassed *IGFALS* (Fig. 2b). Array-based CGH identified no pathogenic copy number variations.

**Fig. 2:**
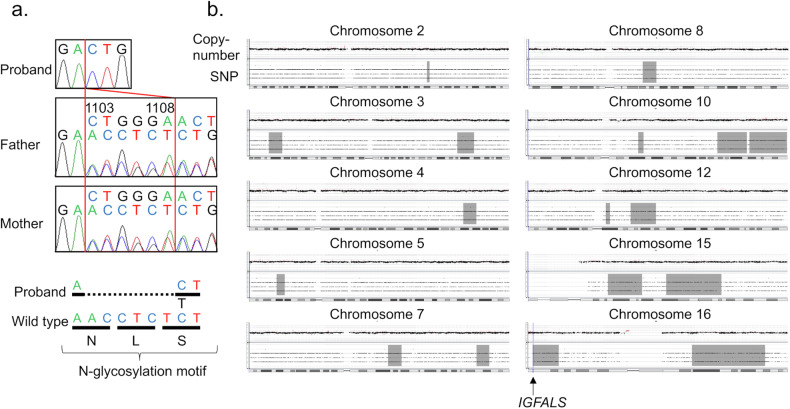
Molecular data of the patient. **a** Sanger sequencing showing the c.1103_1108del, p.N368_S370delinsT variant. The parents of the patient were heterozygous carriers. The 6-bp deletion was located within the fourth N-glycosylation motif (N-L-S). **b** The results of comparative genomic hybridization using a SNP + CGH array. The gray-shaded areas depict regions of loss of heterozygosity (LOH). The LOH region on chromosome 16 included *IGFALS*.

The boy carried a hitherto unreported variant of *IGFALS*. The homozygosity of this rare variant can be ascribed to parental consanguinity; the patient carried large LOH regions on 10 chromosomes. The total size of the LOH regions (187 Mb, 6.05% of the entire genome) was similar to the theoretical size of LOH regions in a child born to first-cousin parents (6.25%)^20^. Of note, the heights of the patient (~−2.6 SD) and his heterozygous parents (−1.0 SD and −1.9 SD) were comparable to those of previously reported individuals with biallelic and monoallelic pathogenic variants of *IGFALS*, respectively (biallelic variants, −2.46 ± 1.07 SD and monoallelic variants, −1.13 ± 1.08 SD)^1^. Moreover, the endocrine data of our patient, such as high GH levels and a low IGF-1 level, were indicative of ALS deficiency^5^. In addition, he had no pathogenic variants in other major growth genes. These results indicate that his phenotype results from the *IGFALS* variant. The patient may develop delayed puberty in the future because this is a common manifestation of ALS deficiency^6^.

The *IGFALS* variant in the patient was a 6-bp deletion. This in-frame deletion is unlikely to cause gross conformational changes or mRNA decay; however, this deletion disrupted one of the seven N-glycosylation motifs of ALS. The phenotype of our patient implies that the loss of the fourth N-glycosylation motif abolishes ALS function. Consistent with this, 3D structure prediction suggested that the asparagine residue at the 368 position mediates the binding between ALS and the binary complex^4^. These results highlight the clinical importance of the N-glycosylation motifs of ALS. In conclusion, this study expands the mutation spectrum of *IGFALS* to include 2-amino acid deletion within an N-glycosylation motif.

## HGV database

The relevant data from this Data Report are hosted at the Human Genome Variation Database at 10.6084/m9.figshare.hgv.3412.

## References

[1] Domené, S. & Domené, H. M. The role of acid-labile subunit (ALS) in the modulation of GH-IGF-I action. *Mol. Cell Endocrinol.* **518**, 111006 (2020). 32861700 10.1016/j.mce.2020.111006

[2] Janosi, J. B., Firth, S. M., Bond, J. J., Baxter, R. C. & Delhanty, P. J. *N*-linked glycosylation and sialylation of the acid-labile subunit. Role in complex formation with insulin-like growth factor (IGF)-binding protein-3 and the IGFs. *J. Biol. Chem.* **274**, 5292–5298 (1999). 10026136 10.1074/jbc.274.9.5292

[3] Firth, S. M., Yan, X. & Baxter, R. C. D440N mutation in the acid-labile subunit of insulin-like growth factor complexes inhibits secretion and complex formation. *Mol. Endocrinol.* **25**, 307–314 (2011). 21177759 10.1210/me.2010-0295 PMC5417311

[4] Kim, H. et al. Structural basis for assembly and disassembly of the IGF/IGFBP/ALS ternary complex. *Nat. Commun.* **13**, 4434 (2022). 35907924 10.1038/s41467-022-32214-2 PMC9338993

[5] Argente, J., Tatton-Brown, K., Lehwalder, D. & Pfäffle, R. Genetics of growth disorders-which patients require genetic testing? *Front. Endocrinol. (Lausanne)* **10**, 602 (2019). 31555216 10.3389/fendo.2019.00602 PMC6742727

[6] Poyrazoğlu, Ş. et al. A novel homozygous mutation of the acid-labile subunit (*IGFALS*) gene in a male adolescent. *J. Clin. Res Pediatr. Endocrinol.* **11**, 432–438 (2019). 30717585 10.4274/jcrpe.galenos.2019.2018.0301 PMC6878349

[7] Castilla-Cortazar I., et al. Growth hormone insensitivity: Mexican case report. *Endocrinol. Diabetes Metab. Case Rep.* **2017**, 17-0126 (2017). 10.1530/EDM-17-0126 PMC5682564 29147569

[8] Plachy, L. et al. High prevalence of growth plate gene variants in children with familial short stature treated with GH. *J. Clin. Endocrinol. Metab.* **104**, 4273–4281 (2019). 30753492 10.1210/jc.2018-02288

[9] Domené, H. M. et al. Heterozygous *IGFALS* gene variants in idiopathic short stature and normal children: impact on height and the IGF system. *Horm. Res Paediatr.* **80**, 413–423 (2013). 24335034 10.1159/000355412

[10] Wit, J. M. et al. Genetic analysis of short children with apparent growth hormone insensitivity. *Horm. Res Paediatr.* **77**, 320–333 (2012). 22678306 10.1159/000338462

[11] Franzoni, A. et al. Novel *IGFALS* mutations with predicted pathogenetic effects by the analysis of AlphaFold structure. *Endocrine* **79**, 292–295 (2023). 36348166 10.1007/s12020-022-03244-z

[12] Grandone, A. et al. Clinical features of a new acid-labile subunit (*IGFALS*) heterozygous mutation: anthropometric and biochemical characterization and response to growth hormone administration. *Horm. Res Paediatr.* **81**, 67–72 (2014). 24356109 10.1159/000355017

[13] Yoo, H. J. et al. Whole exome sequencing for a patient with Rubinstein-Taybi syndrome reveals de novo variants besides an overt CREBBP mutation. *Int J. Mol. Sci.* **16**, 5697–5713 (2015). 25768348 10.3390/ijms16035697 PMC4394500

[14] Kamil, G., Yoon, J. Y., Yoo, S. & Cheon, C. K. Clinical relevance of targeted exome sequencing in patients with rare syndromic short stature. *Orphanet J. Rare Dis.* **16**, 297 (2021). 34217350 10.1186/s13023-021-01937-8 PMC8254301

[15] Kumar, A. et al. Pathogenic/likely pathogenic variants in the *SHOX*, *GHR* and *IGFALS* genes among Indian children with idiopathic short stature. *J. Pediatr. Endocrinol. Metab.* **33**, 79–88 (2020). 31834863 10.1515/jpem-2019-0234

[16] Hattori, A. et al. Next-generation sequencing-based mutation screening of 86 patients with idiopathic short stature. *Endocr. J.* **64**, 947–954 (2017). 28768959 10.1507/endocrj.EJ17-0150

[17] He, J. et al. Preliminary investigation into the genetic etiology of short stature in children through whole exon sequencing of the core family. *Open Life Sci.* **19**, 20220853 (2024). 38737102 10.1515/biol-2022-0853 PMC11087740

[18] David, A. et al. Acid-labile subunit deficiency and growth failure: description of two novel cases. *Horm. Res Paediatr.* **73**, 328–334 (2010). 20389102 10.1159/000308164 PMC2868526

[19] Richards, S. et al. Standards and guidelines for the interpretation of sequence variants: a joint consensus recommendation of the American College of Medical Genetics and Genomics and the Association for Molecular Pathology. *Genet. Med.* **17**, 405–424 (2015). 25741868 10.1038/gim.2015.30 PMC4544753

[20] Sund, K. L. et al. Regions of homozygosity identified by SNP microarray analysis aid in the diagnosis of autosomal recessive disease and incidentally detect parental blood relationships. *Genet. Med.* **15**, 70–78 (2013). 22858719 10.1038/gim.2012.94

